# Microglial diversity by responses and responders

**DOI:** 10.3389/fncel.2014.00101

**Published:** 2014-04-01

**Authors:** Ulla Gertig, Uwe-Karsten Hanisch

**Affiliations:** Institute of Neuropathology, University of GöttingenGöttingen, Germany

**Keywords:** diversity, cytokines, immunity, innate, microglia, subtypes, TLR

## Abstract

Microglia are the principal resident innate immune cells of the CNS. Their contributions to the normal development of the CNS, the maintenance and plasticity of neuronal networks and the safeguarding of proper functionality are becoming more and more evident. Microglia also survey the tissue homeostasis to respond rapidly to exogenous and endogenous threats, primarily with a protective outcome. However, excessive acute activation, chronic activity or an improper adaptation of their functional performance can foster neuropathologies. A key to the versatile response behavior of these cells is their ability to commit to reactive phenotypes, which reveal enormous complexity. Yet the respective profiles of induced genes and installed functions may build up on heterogeneous contributions of cellular subsets. Here, we discuss findings and concepts that consider the variety of microglial activities and response options as being based—at least in part—on a diversity of the engaged cells. Whether it is the production of proinflammatory cytokines, clearance of tissue debris, antigen presentation or the ability to sense neurotransmitters, microglial cells present with an unanticipated heterogeneity of their constitutive and inducible features. While the organizational principles of this heterogeneity are still largely unknown, functional implications are already perceptible.

The term “microglia” is commonly used as a plural word. The tenaciousness by which text processing software marks respective phrases for correction and insists on a singular form may inspire a reflection of the true meaning of “the” microglia as to their plurality. Several and especially most recent observations raise the question whether microglia do represent a uniform entity or a heterogeneous community—with subpopulations being distinguishable by their functional capacities and executed functions (Fitzner et al., 2011; Scheffel et al., 2012; Pannell et al., 2014).

Here we take examples from selected domains of microglia physiology, including monitoring of neuronal activity, synthesis of cytokines, expression of molecules for antigen presentation or the ability for clearing myelin material, to discuss the heterogeneity of microglia in serving housekeeping duties, sensing environmental signals and organizing their (mostly) adequate responses to a disturbed CNS homeostasis. Even though the evidence for such a heterogeneity by equipment (expressed molecules) and performance (function) is ubiquitous, the underlying determinants, the physiological significance and the pathophysiological impact remain largely enigmatic (Hanisch, 2013a,b,c).

## Is there a need for addressing microglial constitution and performance at a single cell level?

The characteristics as neuroimmune cells and the versatile response behavior of microglia upon infection or tissue injury, following to ischemic stroke or in neurodegenerative diseases, as part of an autoimmune disorder or by exposure to a tumor have been described already in detail–with increasing broadness regarding the accompanying molecular profiles and even the dynamics of activity changes (Pukrop et al., 2010; Kettenmann et al., 2011; Prinz et al., 2011; Hickman et al., 2013; Hanisch, 2013a; Butovsky et al., 2014). It is generally understood that a particular challenge within a given context can lead to a distinct reactive phenotype (Hanisch and Kettenmann, 2007; Eggen et al., 2013; Hanisch, 2013a).

Originally based on extraneural macrophages, reactive phenotypes were defined by triggering responses to cytokines and microbial agents, such as interferon-γ (IFNγ) from T helper cell type 1 (Th1) and natural killer cells, interleukin-4 (IL-4) from Th2 cells and Toll-like receptor (TLR) agonists, such as lipopolysaccharide (LPS), a cell wall component of gram-negative bacteria and a widely used standard tool for triggering proinflammatory reactions in myeloid cells. In a simplified concept, exposure to IFNγ or LPS would induce a “classical” activation, also commonly defined as M1 reaction, which comes with the production of proinflammatory mediators in support of defense-oriented Th1 type immune reactions. On the contrary, IL-4 would instruct an “alternative” activation, also termed M2, with a distinct profile of induced genes. This phenotype assists Th2 type immune responses, exerts anti-inflammatory effects, resolves inflammation and supports tissue repair (Hanisch, 2013b). While reciprocal expression of IL-12 and IL-10 served as a first indicator for M1 *versus* M2 commitment, and further molecules were identified with more or less biased association, the sets of regulated genes and functions were found way more complex and not necessarily exclusive. With more activating scenarios being analyzed, it became clear that reactive phenotypes of either M1 or M2 tendencies can vary by the molecular signatures. Attempts of subclassification were very helpful. Major orientations could be defined. Yet a simple polarization by pure M1 *versus* M2 reactions may represent an extreme. Challenges by pathogen-associated molecular patterns (PAMPs) as derived from bacteria or viruses, confrontations with tumors or apoptotic cells, exposure to tissue debris or damage-associated molecular patterns (DAMPs) from impaired cells, a plethora of factors acting in concert simultaneously or in sequential order will tailor adapted macrophage responses. In their variety, they may almost appear as a continuum and cover combinations or simultaneous presence not easily fitting a dual classification system (Sierra et al., 2007; Mosser and Edwards, 2008; Nikodemova et al., 2013). Moreover, reactive phenotypes will also undergo adjustments (Kigerl et al., 2009). Early profiles for defensive actions may shift and deescalate upon successful elimination of the infectious agent or wound closure. At later stages, reactive phenotypes have to mature to promote tissue repair and to support restoration of the structural integrity and—as far as possible—functional recovery.

With the emerging concept of reactive phenotype diversity of macrophages it became also obvious for microglia that distinct stimuli trigger distinct responses (Hanisch and Kettenmann, 2007; Hanisch, 2013c). Defined experimental conditions *in vitro* helped to determine how microbial agents or cytokines lead to an expression of surface receptors and enzymes or to a release of soluble mediators, namely cytokines themselves. Increased motility and directed movements, phagocytic clearance, stimulation of T cell proliferation or impacts on neuronal survival were studied. *In vivo*, a characterization is made more difficult since infiltrating monocytes can intermingle with the resident microglia under various disease conditions and in their respective animal models. Morphological criteria or staining for marker proteins, such as CD11b or Iba1, are not sufficiently reliable, although some distinction can be done by the level of expression, as in the case of CD45. Options for segregating microglia from immune infiltrates have been improved by the introduction of genetically engineered mouse models expressing fluorescent proteins under the control of chemokine receptor promoters, such as for CCR2 and CX_3_CR1 (Mizutani et al., 2012). Elegantly applied in diverse experiments, these studies revealed invaluable insights into the populational maintenance *versus* replenishment or the functional contributions of microglia in health and disease—and to address their versatile reactive behavior (Prinz et al., 2011).

However, in many (if not most) cases, a reactive phenotype is assigned to the affected or responding microglia as a bulk. It appears as an averaged profile. More or less equal contributions of individual cells are assumed to sum up. Measurements of cytokine contents in a culture supernatant or gene array analyses on tissue samples do not detect the parameters at cellular level. Studies with cellular resolution, such as conventional flow cytometry, ELISPOT assays, *in situ* hybridization, PCR analyses on laser capture samples or classical immunocytochemistry, are often restricted by the number of analytes. What if different reactive phenotypes and their prominent features are supported by distinct cells? What if microglial subpopulations take the lead in a response? What if functions are directed to specialized subtypes? These questions sound academic, on a first glance. They gain value when inspecting (and trying to interpret) the enormous complexity, overlaps and dynamics of phenotypic orientations and when considering targets and tools for potential (therapeutic) manipulations. Ideally, multiparametric information on both the molecular equipment and their functional translations should be available for circumscribed populations or individual cells in combination with their local position and changes over time.

For neurons, it is text book knowledge that subtypes exist by morphology, neurotransmitter use, coexpressed molecules and electrophysiological parameters. Distinctions are made also for astrocytes (Emsley and Macklis, 2006; Matyash and Kettenmann, 2010). For immune cells, like T cells, subsets are defined by expression and release properties with direct importance for supported immune functions. Still single-cell technologies are on the verge to be required—and to be at hand—for covering substantial contributions of rare subsets and to explore the individual phenotypic variability for identified cells at a given time point and with kinetic resolution (Shalek et al., 2013; Chattopadhyay et al., 2014).

## How does information about the category microglia explain the function of a cell?

Microglia comprise a rather large population. By parenchymal localization in the brain, spinal cord as well as the retina, they differ from other myeloid cells within the cranial compartment, such as those in the meninges, the choroid plexus or the perivascular space (Ransohoff and Cardona, 2010; Kettenmann et al., 2011; Prinz et al., 2011). CNS regions vary by microglial density, morphology and expression of a substantial number of gene products, even though physiological implications are still barely addressed. Heterogeneity in cell shape and the constitutive or episodic expression of certain molecules has been noticed since a long time (Streit and Graeber, 1993), but not necessarily and always with a conclusive interpretation as to cellular subsets (Kettenmann et al., 2011; Hanisch, 2013b).

Recently, regional differences have been addressed more consciously and comprehensively by looking at expression profiles and associated functional properties, distinct responsiveness to activating stimuli and adjustments that occur with development, aging or diseases, whereas information on gender differences or the comparability between species is still scarce (McCluskey and Lampson, 2001; Sierra et al., 2007; de Haas et al., 2008; Lai et al., 2011, 2013; Hart et al., 2012; Nikodemova et al., 2013; Torres-Platas et al., 2014). Enormous efforts have now been taken to characterize at large scale the transcriptome and microRNA spectrum of microglia isolated from newborn, adult and aged mice, in comparison to other major CNS cell types, other immune cell populations or the human microglia counterpart—thereby not only delivering exhaustive data sets but also extracting microglial signatures (Hickman et al., 2013; Butovsky et al., 2014). The findings build up on and extend previous lines of research that demonstrate the uniqueness of microglia among the family of tissue macrophages as to their lineage origin within the mononuclear phagocytic system, as to their self-maintenance as well as properties acquired and exhibited within the context of their tissue environment (Ginhoux et al., 2010; Gautier et al., 2012; Schulz et al., 2012; Davies et al., 2013; Greter and Merad, 2013; Kierdorf et al., 2013; Wynn et al., 2013).

Accordingly, microglia are distinguished as a class of cells. Tissue-architectural, cellular and biochemical specificities of the CNS regions may impose further adaptations. Yet particular actions as a helper in development, as a housekeeper and sentinel in homeostatic surveillance or as an innate immune cell in fighting infection and endogenous threats have to be organized by an individual cell in contact with its immediate neighborhood, which may instruct and require adapted microglial properties.

## How much individuality can fit into the current concepts of a microglial cell?

A distinction of microglia could be based on single proteins that are sufficiently different at constitutive expression levels or unequally induced on demand—or by combination of both if there is some coincidental or causal link. Microglia differing by CD40 expression reveal concomitantly either the ability or inability for the induction of inducible nitric oxide synthase (iNOS) and release of tumor necrosis factor (TNF) α upon stimulation with LPS (Kawahara et al., 2009). Classification by the presence *versus* absence of a “marker” does not yet reflect individuality. However, when features with dichotomic expression are combined the variety of patterns increases quickly.

**Figure 1:**
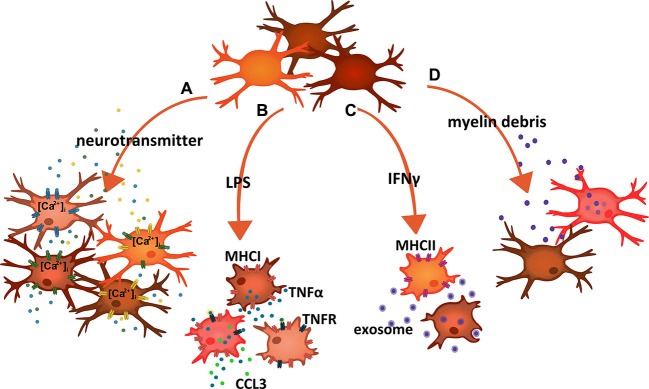
**Schematic summary of examples indicating microglial response heterogeneity. (A)** Stimulation of microglia cells with neurotransmitters and neurohormones triggering calcium signals revealed that only small fractions responded to each compound. Combined stimulation and population analysis thereby suggested an enormous variety in terms of functional receptor expression. **(B)** Exposure to LPS induced a panpopulational expression of major histocompatibility complex I (MHCI) molecules, indicating that all microglia expressed TLR4. In contrast, only subsets also produced TNFα and/or CCL3. **(C)** Treatment of microglia with IFNγ caused the expression of major histocompatibility complex II (MHCII) in some but not all cells. On the other hand, MHCII-expressing microglia did not clear myelin-laden exosomes, a function that rather associated with MHCII-negative cells. **(D)** Exposure of cells to myelin debris resulted in the phagocytic uptake of the material in a subset of microglia. Adapted and expanded from Scheffel et al. (2012).

Microglia express a range of neurotransmitter receptors (Pocock and Kettenmann, 2007; Kettenmann et al., 2011). Neuronal activity can thereby influence their migratory behavior, phagocytic activities or the release of cytokines, chemokines and other mediators. Impaired neuron-to-microglia signaling can cause severe dysregulations of microglial functions during development, as part of the aging process and, of course, in neurodegenerative diseases. In turn, abnormal microglia impacts on neuronal cells and their circuitry (Heneka et al., 2010; Zhan et al., 2014). Even though neurons can exert influences on microglia by a variety of ligand-receptor systems, including CD200/CD200R, SIRP1α/CD47, CX_3_CL1/CX_3_CR1 or CD22/CD45 (Kettenmann et al., 2013; Hanisch, 2013b), also classical neurotransmission could play critical roles in organizing daily surveillance and maintenance activities as well as controlling of innate immune actions upon a challenge. A just published study systematically investigated the functional expression of numerous neurohormone and neurotransmitter receptors in mouse microglia (Pannell et al., 2014). Not only does this work reveal richness in the sensory spectrum. It indicates an unanticipated heterogeneity as well (Figure 1A).

Following up former work (Seifert et al., 2011), and based on inducible calcium signals recorded with fluo-4, the study addressed the sensitivity of acutely isolated adult as well as cultured neonatal and adult mouse microglia to a panel of substances. Cells were exposed to angiotensin II, dopamine, endothelin, galanin, histamine, neurotensin, nicotine, serotonin, somatostatin, substance P or vasopressin at relevant concentrations. Only subpopulations responded to a given compound with a calcium transient, in most cases less than 20% of the cells. The subpopulational responses were similarly observed in the three preparations. Thus, and along with other technical aspects, isolation or culture artifacts could be ruled out. Pre-treatment with agents inducing M1 (LPS and IFNγ) or M2 (IL-4) polarization led to individual increases and decreases in the size of the responder populations. While the patterns may vary with the age of the animals at which cells were harvested and with the influence of modulating factors, the essential message is diversity among cells. Combinatory delivery (by a consecutive treatment) of neurotransmitters/neurohormones led then to the conclusion that the respective receptor expressions are largely at random and that microglia present with an immense diversity.

Of course, isolation of microglia from larger brain parts for a study *in vitro* cannot correlate the response pattern to anatomical divisions. Features found for subsets may relate to the need for local communication with neurons and neurosecretory cells in a cortical layer or a nucleus. In this regard, and as an example of a compound included in the screening, substance P was found to exert distinct effects on the IFNγ-induced expression of MHCII molecules in the brainstem *versus* the hippocampus of rats, ranging from strong enhancement to very little (if any) impact (McCluskey and Lampson, 2001). Yet culture conditions as applied by Pannell and coworkers do not seem to override these adaptations and the functional receptor expression may not reside only in one subset (Pannell et al., 2014). Of course, this study cannot inform about (each or even a global) functional implication, but it estimates an organizational distinction among microglia at a level far above any differentiation based on single molecule expression (Kettenmann et al., 2011; Hanisch, 2013b). Correlations with functions are still more limited by number. Yet the examples at hand could further substantiate the understanding of “the” microglia as a generic term for a diversified cell community.

## Can discrete subsets of microglia be assigned to particular functions?

While the above example displays an enormous spectrum of microglia by sensory equipment, activation of a single receptor can also lead to distinct responses in subsets. TLR4 is known for recognizing LPS as a PAMP, but it also mediates responses to a range of disparate self-derived molecules acquiring the meaning of a DAMP in noninfectious settings (Hanisch, 2013a,c). Among its family members, TLR4 is the most complex by signaling options and interactions with other receptor and co-receptor proteins. We had reported that its function in mouse microglia undergoes a massive change during postnatal development, apparently correlating with some reorganization in signaling pathways, but also showing a learning process in the functional interpretation of structural LPS variants (Regen et al., 2011; Scheffel et al., 2012).

Stimulation of mouse microglial TLR4 triggered the panpopulational upregulation of MHCI, a surface structure required for antigen presentation to cytotoxic T cells. As expected, TLR4 activation also led to the release of TNFα. However, when resolved at single cell level, only a subpopulation carried this activity (Figure 1B). The demarcation of the subpopulation got sharper with increasing postnatal age of the mice. Including also CCL3 (MIP-1α) as a T cell-attracting chemokine in the analysis, the TNFα^+^ microglia subpopulation could be further separated into cells with a combined production and those only producing TNFα (Figure 1B). Focusing on CNS regions, the subsets were found in the cerebral cortex, the cerebellum and the spinal cord. With a delivery of the pleiotropic and proinflammatory cytokine relying on a subset and responses being imposed on many more cells expressing TNF receptors (including other microglia and CNS-resident as well as invading immune cells), a principle of “master instruction” could be established, in which a small set of TNFα-producing microglia takes an essential role in coordinating tissue responses (Clausen et al., 2008; Lambertsen et al., 2009). It is important to note that the detection of a subpopulation responding to TLR4 stimulation with TNFα cannot be explained by unequal TLR4 expression (Scheffel et al., 2012). Almost all cells showed TLR4-mediated induction of MHCI. Organization of a discrete production of the cytokine is thus an inherent property of the TLR4 signaling cascade in some but not all cells. Notably, selective responses can also be triggered through other receptor systems.

IFNγ can trigger the expression of MHCII structures for professional antigen presentation. Microglia respond in this way both *in vitro* as well as *in vivo* (Fitzner et al., 2011; Figure 1C). Different regions of the CNS thereby reveal a different response sensitivity and/or induction capacity (McCluskey and Lampson, 2001), and it is again only a subset of microglial cells that present with a detectable expression (Fitzner et al., 2011). A subset-dependent expression is also seen for the costimulatory molecules CD80 and CD86, which act in support of MHCII function. A demonstration of heterogeneous MHCII induction in microglia upon IFNγ injections into the CNS also considered potential gradient effects, which may feignedly reveal MHCII^-^ microglia in vicinity to MHCII^+^ cells, but just because of insufficient stimulation. Nevertheless, the MHCII^+^ cells were rather scattered and blended with those lacking any staining signal. Moreover, pathohistology data from human postmortem material corroborated the findings in rodents as to a restricted microglial expression of respective human leucocyte antigen molecules. Apparently, not all microglia would be able to act as an antigen-presenting cell (APC). Heterogeneity of microglia as to APC functions has been noticed also on the basis of CD11c expression and actual performance (Remington et al., 2007). Interestingly, intracellular MHCII molecules may also serve some additional and previously unknown function in TLR4 signaling (Liu et al., 2011). This link could integrate in the subset organization under TLR4 activation (Hanisch, 2013b).

The very same study addressed the activity of microglia to clear myelin-laden exosomes from oligodendrocytes, under normal conditions and as a housekeeping function in support of myelin turnover (Fitzner et al., 2011; Figure 1C). This would well fit to the notion of microglia as never-resting sentinels and servants, which continuously scan their environment, but also “nurse” and shape synaptic connections (Davalos et al., 2005; Nimmerjahn et al., 2005; Haynes et al., 2006; Wake et al., 2009; Graeber, 2010; Paolicelli et al., 2011; Tremblay et al., 2011; Schafer et al., 2012; Kettenmann et al., 2013; Zhan et al., 2014). The exosome removal, as performed by macropinocytosis, associated with some but not all cells. Surprisingly, when combining a detection of the MHCII surface structures upon IFNγ treatment with an analysis of exosome uptake, populations were largely exclusive, meaning that MHCII^+^ microglia would not be concerned with myelin clearance and *vice versa*. This principle was, therefore, termed immunologically silent myelin removal (Fitzner et al., 2011). Thought to the end, it suggests a compartmentalization where self-derived material destined for degradation is sequestered from a pool of cells which can present material to the adaptive immunity for appropriate attack. Here, two subpopulations exercise a division of labor, probably to avoid a dangerous collision of functions. Conceivably, an accidental spill of myelin material into the cellular domain potentially serving APC functions could come with a risk of autoimmune responses, as they occur in multiple sclerosis.

Microglia can remove myelin debris resulting from damage and autoimmune destruction, an essential contribution to allow for repair and to reduce further damage (van Rossum et al., 2008; Gitik et al., 2011; Hadas et al., 2012). Uptake of myelin material is preferentially seen in a subpopulation of cells (Regen et al., 2011; Scheffel et al., 2012; Figure 1D). Similar to the disposal of the exosome-wrapped myelin, phagocytosis of myelin debris is suppressed by TLR4 activation, although the mechanisms of incorporation *per se* significantly differ from each other. On the other hand, activity of TLR signaling can rather enhance clearance of pathogens, such as Gram-negative and –positive bacteria (Ribes et al., 2009, 2010), a function that also appears to be associated with microglial subsets. It would be very interesting to determine such a subpopulational split for other clearance cargo as well.

Taking together, various lines of evidence combine in a notion of microglial diversity by both spontaneous and inducible functions. Such a concept could have multiple implications. Thus far, heterogeneity with regard to microglia has been mainly seen in the light of different reactive phenotypes, i.e., diversity by expressed genes and activities. Underlying organization principles and the contributions by individual cells were mostly neglected or not explicitly scrutinized. A split into subsets for a single protein expression or a given task can repeatedly be observed in many cases. Their comprehensive compilation, however, would probably give a complex matrix of microglial activity and response options. Anatomical resolution would be required to sort such subsets to CNS regions.

Some features and feature combinations may concentrate in some brain structures and may not be distributed throughout others, such as the proliferative potential (Walton et al., 2006; Marshall et al., 2008; Thored et al., 2009). Cells in the subventricular zone were found to be distinguished by their proliferative capacity and the support of neurogenesis. Proliferative potential would be important for the self-renewal of microglia, but might be a function especially carried out by some cells (Gomez Perdiguero et al., 2013). Similarly, support of neurogenesis and oligodendrogenesis, as a demonstrated activity of microglia (Butovsky et al., 2006), could be organized in some (functional) niches. How more locally situated cells could deploy and offer their specialized functions at other sites is not quite obvious. Even though microglia use their motile processes for scanning, they are rather stationary, at least under normal conditions. On the other hand, they can migrate on demand.

While microglia may have distinct capacities among themselves they differ from monocytes. Infiltration of peripheral immune cells in CNS pathologies could be essential whenever some requested know-how is simply not offered by microglia, not even by their specialized subsets. In such situations, the CNS may depend on extraneural monocytes/macrophages. What kind of expertise would then not be properly covered by the microglial spectrum? Certain aspects of phagocytosis, assistance in certain immune functions? However, these question cannot be answered satisfyingly right now—and it is also unclear whether all the observed heterogeneity by subsets has a fundament in truly distinct subtypes of microglia. In other words, how would individual microglial cells acquire distinct functional and reactive behavior?

## How could functional diversity of microglia be installed?

Various mechanisms could be envisaged. As major and fundamental—but still hypothetical—alternatives for an explanation one may consider a rather stable installation of differences in “subtypes” or one that is more instructed by environmental cues. Still another theory could be based on an entirely stochastic process.

In series of seminal studies, the ontogenetic origin and the maintenance of a stable microglia population have been dissected, identifying sources, critical transcription factors and steps of microgliogenesis (Naito et al., 1996; Ginhoux et al., 2010; Gautier et al., 2012; Gomez Perdiguero et al., 2013; Schulz et al., 2012; Yona et al., 2012; Kierdorf et al., 2013). It is speculative to assume that dissection of lineage origin and stages could still leave room for a late split into cells that later give rise to functionally distinct microglia subtypes. If so, how many distinct versions could be generated to accommodate the variety of microglial responses by distinguished responders? How and when would they be distributed throughout the CNS tissues? Such “hardware-based” concepts may quickly run into a dead end since it would imply too many bifurcations and too much of a logistic effort.

Instructions of microglial cells by the (micro) environment appear to be more feasible. The cellular neighborhood, the extracellular matrix (ECM) or features of the blood-brain barrier as varying by CNS region could impose a myriad of soluble and immobilized cues and signals to organize the properties of microglia (Hanisch, 2013b). The ECM is seen as a provider of compartments and as an organizer of functional microdomains (Dityatev et al., 2010). Factors circulating in the blood may have restricted entry to the CNS parenchyma at particular sites of the CNS, where the local microglia expresses respective sensor molecules (Perry et al., 1992). Heterogeneity of microglia may go hand in hand with the heterogeneity of other glial cells (Emsley and Macklis, 2006; Kitada and Rowitch, 2006; Matyash and Kettenmann, 2010). The prevailing neurotransmitter in a cortical layer or a nucleus may govern microglial assets as to receptors as much as microglial cells participate in the maturation of synaptic connectivity (Parkhurst et al., 2013; Zhan et al., 2014). Instruction by multiple inputs at a specific location could offer advantages for explaining microglial heterogeneity. First, it would define cellular properties locally, and not beforehand for a whole set of microglia which still would need to be properly placed. Second, combination of numerous instructing signals—rather than a single “master” factor—could then also better organize a range of distinct features. Microglia themselves may take influences. At a lesion site, attracting microglia and causing higher than normal cell density, profiles of cytokines and chemokines could result from unequal release activities as organized by cooperative action. As stressed above, some cells may take the lead. Early production of key factors by a cellular subset could subsequently govern the responses of others. Distinct specializations and respective subset sizes might be precisely arranged under such conditions to adapt to the actual needs.

We have shown that microglia undergo a postnatal maturation process, regarding signaling properties of TLRs and including the ability of TLR4 to distinguish between PAMP ligand variants as to inducible cytokine profiles (Scheffel et al., 2012). In mice, a window between postnatal days P21 and P49 was thereby found to be critical. The period coincides with other CNS maturation aspects, including the formation of myelin sheets. The fact that and also how microglial maturation continues after birth has been addressed in a recent study, showing that the period between P21 and about P60 comes with a strong expression of genes that define the signature of (adult) microglia (Butovsky et al., 2014). This work also identified transforming growth factor (TGF) β as a key molecule in this process.

Features acquired within a defined environment during a particular developmental window may either remain stable or stay subject to adaptations and re-instruction. This could apply to microglia in general as well as to their potential subsets. It would be interesting to test for the stability of regional properties by exchanging local populations and determining characteristic features in a new environment. The observation that microglia can be harvested at given developmental stages and kept *ex vivo* with distinct features may suggest that they preserved at least some of the properties acquired within the tissue (Scheffel et al., 2012; Pannell et al., 2014).

How long would a regional imprint last without renewal by the critical cues? Could cells also change properties upon exposure to key signals or following a drastic activation? We had recently discussed issues of the postactivation fate of microglia in which experience of a previous challenge may alter responses to a subsequent encounter of the same or a different stimulus (Hanisch, 2013a,b). Epigenetic mechanisms could provide the molecular script for such “learning” processes. The long-lived microglia may also accumulate over the years (and decades) traces of repeated minor challenges and thus slowly alter surveillance or response properties in the aging CNS. Some diversity may thereby build up also with time, rendering some cells less efficient in providing support for maintenance, or yielding cells which do not properly respond to calming and activating signals anymore. Instructed during normal development or by episodic influences, diversity among microglia would in all these cases rely on a preserved assignment of particular functions to certain cells. However, a solution for the question of how microglia reveal distinct performance could also be found in a principle not depending on any determination at all.

Subpopulations as to the induction of a gene could be “created” by chance, resulting from a stochastic process of transcription (Hume, 2000). Variable stability of mRNA in cells and the consequence for the respective translation is a key in this concept, which explicitly applies to leukocyte differentiation and activities. It could also explain heterogeneity in TNFα synthesis upon TLR4 stimulation (Ravasi et al., 2002). The tempting and data-supported principle could thus help saving efforts in identifying complex instructions. Environmental factors as well as intrinsic mechanisms as based on probability may cooperate to create a situational response diversity, as transcriptional regulation in macrophages still seems to be complex (Lawrence and Natoli, 2011). Microglia may leave the synthesis of an important cytokine to a subset by chance, as long as it is secured that some cells will “feel responsible”. Yet it would be more difficult to imagine a complex interaction of numerous gene products, as for example needed for antigen processing and presentation. Genes with grouped functions might be regulated *en bloc*, but would a stochastic process suffice a timed and synergistic performance? What about the heterogeneity observed for microglial responses to neurotransmitters and neurohormones (Pannell et al., 2014)? Is the respective receptor equipment expressed ubiquitously and at random or in correlation to the neuronal community? Would a sufficiently strong stimulation of a receptor with panexpression in microglia (like TLR4) but still triggering a response in only some of them (like the production of TNFα) at one point drive the induction in the entire population? Or would the competence for it remain a subset feature?

Regardless of which explanation will turn out to be suitable, the diversity in microglial actions remains as a phenomenon. It may stay a subordinated aspect in the organization of reactive phenotypes or an annotation when deciphering housekeeping functions. On the other hand, it could provide a clue to the understanding of the multi-faceted activities of microglia taking place on a daily basis throughout the CNS and in situations of emergencies.

## Conclusion

There is virtually no neuropathological event which remains unnoticed by microglia. Their involvement is indicated by signs of “activation”. The term, however, does not tell much about the actual activities (Hanisch and Kettenmann, 2007). Microglia may themselves be part of the pathogenetic process or rather attempt to contain its aggravation. Failure in protection would be detrimental. Excessive acute, sustained (chronic) activity or improperly adjusted reactive phenotypes may also render microglia a disease-driving element. On the other hand, and in the past, there has been no CNS disease declared to be directly and solely linked to a microglial defect, in terms of a cell-specific gene mutation or a selective functional deficit. More recent studies, however, provide strong arguments for a fundamental importance of microglia in normal development. They reveal how disturbances in cellular communication as well as executive functions of microglia could underly impairment of even higher CNS activities and even identify essential gene products therein (Chen et al., 2010; Derecki et al., 2012; Parkhurst et al., 2013; Zhan et al., 2014). Maybe, a massive failure of microglia has an early fatal outcome. Other essential contributions, later in life and serving the homeostasis on a daily basis, are probably missed because they are mostly effective. Many of the minor, locally restricted and transient insults to neural cells may never surface with symptoms due to a rapid protective engagement of microglia. It is probably a subtle deficiency in a particular microglial activity which can then build up to a harmful consequence. It is thus of foremost relevance to dissect the pervasive performance and the emergency measures of microglia—in their duality as a major CNS as well as immune cell and with subpopulational resolution by and within regions.
